# Long non‐coding RNA *CASC8* polymorphisms are associated with the risk of esophageal cancer in a Chinese population

**DOI:** 10.1111/1759-7714.13612

**Published:** 2020-09-02

**Authors:** Yonghua Sang, Haiyong Gu, Yongbing Chen, Yijun Shi, Chao Liu, Lu Lv, Yifeng Sun, Yongsheng Zhang

**Affiliations:** ^1^ Department of Thoracic and Cardiovascular Surgery The Second Affiliated Hospital of Soochow University Suzhou China; ^2^ Department of Thoracic Surgery, Shanghai Chest Hospital Shanghai Jiaotong University Shanghai China; ^3^ Department of Cardiothoracic Surgery Affiliated People's Hospital of Jiangsu University Zhenjiang China; ^4^ Department of Pathology The Second Affiliated Hospital of Soochow University Suzhou China

**Keywords:** *CASC8*, esophageal cancer, lncRNA, polymorphisms

## Abstract

**Background:**

Esophageal cancer (EC) is an important disease that threatens public health and safety. Although there are numerous treatment options for esophageal cancer including surgery, radiation therapy, and chemotherapy, these treatments have limited effects. Its morbidity and mortality vary widely among countries and regions. Esophageal cancer is classified into squamous cell carcinoma (ESCC) and esopheageal adenocarcinoma (EADC). Here, we examined the genetic susceptibility to ESCC in relation to functional single nucleotide polymorphisms (SNPs) in the long non‐coding RNA (lncRNA) *CASC8*.

**Methods:**

To detect the susceptibility to ESCC in relation to functional polymorphisms in *CASC8*, a hypothesis‐driven study was performed to identify *CASC8* SNPs in 949 patients with ESCC and 1369 control subjects.

**Results:**

The *CASC8* rs1562430 GG genotype was significantly associated with increased ESCC risk in men, patients younger than 63 years, non‐smokers, and nondrinkers.

**Conclusions:**

*CASC8* rs1562430 A > G may cause susceptibility to ESCC and *CASC8* SNPs may play a vital role in ESCC risk, thereby serving as a potential biomarker for diagnosing ESCC. A larger sample size and multifactor information are needed to confirm these results.

## Graphical abstract

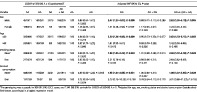

## Introduction

Esophageal cancer, an important threat to the normal function of human digestion, ranks sixth among causes of cancer death.^1^, ^2^ There are various subtypes of esophageal cancer such as adenocarcinoma and squamous cell carcinoma.^3^, ^4^ The incidence and mortality of esophageal cancer ranks eighth and sixth in all malignant tumors in the world; approximately 456 000 new cases of esophageal cancer are diagnosed each year, and this malignancy has a severe effect on people's lives and health.^5^ Despite the use of multimodal therapy, the incidence of esophageal cancer continues to increase, which is mostly attributed to its complex pathogenic mechanism.^6^ Most researchers believe that esophageal cancer is a multifactorial tumor. Biological factors, lifestyle, habits, and environmental factors may be related to the occurrence of esophageal cancer.^7^, ^8^, ^9^

Although the human genome has 3 billion DNA base pairs, only 1.5% of the genome contains coding DNA.^10^, ^11^ However, the remaining non‐coding regions play vital regulatory roles. Most disease‐related single nucleotide polymorphisms (SNPs) occur in non‐coding regions,^12^ and many of these SNPs are associated with cancer. Polymorphisms in many genes including long non‐coding RNAs (lncRNAs) are closely related to tumorigenesis and tumor development.^13^, ^14^ LncRNA is a type of non‐coding RNA that does not participate in protein coding and has a sequence length of >200 nucleotides. Many studies have investigated the correlation between lncRNAs and the pathogenesis of various diseases,^15^ including tumors,^16^, ^17^ and the results suggest that SNPs in lncRNA are associated with the susceptibility to cancer.^18^, ^19^, ^20^

Cancer susceptibility candidate 8 (CASC8) is an lncRNA with no protein‐coding potential that is located in the 8q24 region.^21^ LncRNAs originating from the 8q24 region including CASC8 play a critical role in the regulation of MYC, which is important for the development of multiple tumors, and the expression of *CASC8* is regulated by long‐range interaction of the MYC enhancer with the *CASC8* promoter.^22^

SNPs in the *CASC8* gene, such as rs7837328, rs6983267, and rs7014346, are correlated with the risk of cancer, including prostate,^23^ breast, colorectal, and gastric cancers.^21^ The rs10505477, located in the intron of *CASC8*, is associated with the risk of colorectal cancer^24^, ^25^, ^26^ and the prognosis of gastric cancer.^21^ However, the effect of *CASC8* SNPs on esophageal squamous cell carcinoma (ESCC) remains unclear. Therefore, we performed a hypothesis‐driven study to assess the molecular mechanisms associated with functional *CASC8* SNPs in ESCC.

## Methods

### Study subjects

The current study was confirmed and approved by the Review Board of Jiangsu University (Zhenjiang, China). Written informed consent was obtained from all participants. The study enrolled 949 patients from the Affiliated People's Hospital of Jiangsu University and Affiliated Hospital of Jiangsu University (Zhenjiang, China) between October 2008 and June 2013. A total of 1369 normal controls were selected from the above hospitals during the same time period and frequency‐matched to the patients with respect to age (± 5 years) and sex. Information for each participant was collected through a questionnaire, including information on drinking, smoking, age, sex, and diet. Venous blood (2 mL) was collected from each participant for *CASC8* genotyping.

### Polymorphism genotyping

Genomic DNA samples were isolated using the QIAamp DNA Blood Mini Kit (Qiagen, Berlin, Germany)^27^ and the extracted DNA sample was amplified by polymerase chain reaction (PCR). Hi‐SNP high‐throughput genotyping methods^28^ were used to genotype the amplification products (Shanghai Biowing Applied Biotechnology CO. LTD, Shanghai, China).

### Statistical analysis

Hardy‐Weinberg equilibrium for each SNP in the control subjects was detected using the chi‐squared test. Student's *t*‐tests and χ^2^ tests were performed to detect differences in factors collected in the questionnaire and *CASC8* rs10505477 C > T and rs1562430 A > G genotypes. The relationships between *CASC8* rs10505477 C > T and rs1562430 A > G SNPs and risk of ESCC were examined by computing odds ratios (ORs) and 95% confidence intervals (CIs) using logistic regression analyses, including crude ORs and adjusted ORs, after adjusting for age, sex, smoking, and drinking status. All statistical analyses were performed using SAS 9.1.3 (SAS Institute, Cary, NC, USA).

## Results

### Patient characteristics

The distribution of the demographic characteristics of the 949 cases and 1369 normal controls is shown in Table 1. Statistical analysis showed no significant difference in age or sex between the two groups (*P* = 0.167 and *P* = 0.107). However, the cases group included a significantly higher number of smokers and drinkers (both *P* < 0.001), suggesting that smoking and drinking are related to the development of ESCC. Minor allele frequencies (MAFs) in the controls were similar to East Asian MAFs in the 1000 Genomes database for these SNPs (Table 2).

**Table 1:** Distribution of selected demographic variables and risk factors in ESCC cases and controls

	Cases (*n* = 949)		Controls (*n* = 1369)		
Variable	n	%	n	%	*P*‐value^†^
Age (years) mean ± SD	62.56 (± 8.60)		62.04 (± 9.09)		0.167
Age (years)					0.096
<63	472	49.74	729	53.25	
≥63	477	50.26	640	46.75	
Sex					0.107
Male	659	69.44	907	66.25	
Female	290	30.56	462	33.75	
Tobacco use					**<0.001**
Never	547	57.64	947	69.17	
Ever	402	42.36	422	30.83	
Alcohol use					**<0.001**
Never	666	70.18	1073	78.38	
Ever	283	29.82	296	21.62	

^†^ Two‐sided *χ* ^*2*^ test and student's *t*‐test; Bold values are statistically significant (*P* < 0.05).

**Table 2:** Primary information on *CASC8* rs10505477 C > T and rs1562430 A > G polymorphisms

Genotyped SNPs	*CASC8* rs10505477 C > T	*CASC8* rs1562430 A > G
Chromosome	8	8
Gene official symbol	CASC8	CASC8
Function	Intron variant	Intron variant
Chr Pos (GRCh38.p12)	127 395 198	127 375 606
Regulome DB score^†^	5	5
TFBS^‡^	—	—
Splicing (ESE or ESS)	—	—
miRNA (miRanda)	—	—
miRNA (Sanger)	—	—
nsSNP	—	—
MAF^§^ for East Asian in database (1000 Genomes)	0.389	0.177
MAF in our controls (*n* = 1369)	0.410	0.163
*P*‐value for HWE^¶^ test in our controls	0.265	0.019
Genotyping method	Hi‐SNP	Hi‐SNP
% Genotyping value	97.37%	97.80%

^†^ www.regulomedb.org/.

^‡^ TFBS, transcription factor binding site (http://snpinfo.niehs.nih.gov/snpinfo/snpfunc.html).

^§^ MAF, minor allele frequency.

^¶^ HWE, Hardy‐Weinberg equilibrium.

### Associations between ***CASC8*** rs10505477 C > T and rs1562430 A > G polymorphisms and risk of **ESCC**

As shown in Table 3, the genotype frequencies of *CASC8* rs1562430 A > G were 69.65% (AA), 26.99% (AG), and 3.34% (GG) in the cases and 69.12% (AA), 29.08% (AG), and 1.78% (GG) in the healthy controls, demonstrating statistically significant differences between the two subgroups (*P* = 0.042). In a recessive model using *CASC8* rs1562430 AA/GG genotypes as the reference group, the GG homozygous genotype (GG vs. AA/GG: adjusted OR = 2.11, 95% CI: 1.22–3.64, *P* = 0.007) was significantly associated with increased risk of ESCC, whereas the AG/GG homozygous genotype (AG/GG vs. AA/GG: adjusted OR = 0.97, 95% CI: 0.81–1.16, *P* = 0.725) was not associated with ESCC risk. However, when the *CASC8* rs1562430 AA homozygous genotype was used as the reference group, the GG genotype was significantly associated with increased risk of ESCC (GG vs. AA: adjusted OR = 2.05, 95% CI: 1.18–3.55, *P* = 0.010), whereas the AG genotype was not associated with ESCC risk (AG vs. AA: adjusted OR = 0.91, 95% CI: 0.75–1.09, *P* = 0.304). The *CASC8* rs10505477 C > T SNP was not associated with ESCC risk (Table 3).

**Table 3:** Logistic regression analyses of associations between *CASC8* rs10505477 C > T and rs1562430 A > G polymorphisms and risk of ESCC

	Cases (*n* = 949)			Controls (*n* = 1369)					
Genotype	n	%		n	%	Crude OR (95% CI)	*P*‐value	Adjusted OR^†^ (95% CI)	*P*‐value
*CASC8* rs10505477 C > T									
CC	317	34.05		471	35.52	1.00		1.00	
CT	439	47.15		622	46.91	1.05 (0.87–1.27)	0.620	1.03 (0.85–1.24)	0.800
TT	175	18.80		233	17.57	1.12 (0.88–1.42)	0.375	1.10 (0.86–1.41)	0.437
TT vs. CT vs. CC							0.670		
CT + TT	614	65.95		715	64.48	1.07 (0.90–1.27)	0.471	1.05 (0.88–1.25)	0.622
CC + CT	756	81.20		1198	82.43	1.00		1.00	
TT	175	18.80		233	17.57	1.06 (0.85–1.34)	0.597	1.07 (0.85–1.36)	0.550
C allele	1234	57.63		1812	58.97				
T allele	636	42.37		846	41.03				
*CASC8* rs1562430 A > G									
AA	645	69.65		927	69.12	1.00		1.00	
AG	250	26.99		390	29.08	0.92 (0.76–1.11)	0.393	0.91 (0.75–1.09)	0.304
GG	31	3.34		24	1.78	**1.86 (1.08–3.19)**	**0.025**	**2.05 (1.18–3.55)**	**0.010**
GG vs. AG vs. AA							**0.042**		
AG + GG	281	30.34		414	30.87	0.98 (0.81–1.17)	0.789	0.97 (0.81–1.16)	0.725
AA+AG	895	96.65		1317	98.21	1.00		1.00	
GG	31	3.34		24	1.78	**1.90 (1.11–3.26)**	**0.020**	**2.11 (1.22–3.64)**	**0.007**
A allele	1540	83.15		2244	83.66				
G allele	312	16.84		438	16.33				

^†^ Adjusted for age, sex, smoking status and alcohol consumption; bold values are statistically significant (*P* < 0.05).

### Stratified analyses of associations between ***CASC8*** polymorphisms and **ESCC** risk

Stratified analysis was performed to further assess the possible correlation between the *CASC8* rs1562430 A > G SNP and ESCC risk in the recessive model (Table 4). The results showed that the *CASC8* rs1562430 GG genotype was significantly associated with increased risk of ESCC among men (GG vs. AA: adjusted OR = 2.47, 95% CI: 1.27–4.81, *P* = 0.008), patients younger than 63 years (GG vs. AA: adjusted OR = 2.50, 95% CI: 1.26–4.95, *P* = 0.009), non‐smokers (GG vs. AA: adjusted OR = 1.98, 95% CI: 1.05–3.73, *P* = 0.034), and nondrinkers (GG vs. AA: adjusted OR = 1.98, 95% CI: 1.07–3.66, *P* = 0.031) (Table 4). In addition, in a recessive model using *CASC8* rs1562430 AA/GG genotypes as the reference group, the GG homozygous genotype was significantly associated with increased risk of ESCC among men (GG vs. AA/GG: adjusted OR = 2.62, 95% CI: 1.35–5.10, *P* = 0.005), patients younger than 63 years (GG vs. AA/GG: adjusted OR = 2.62, 95% CI: 1.33–5.17, *P* = 0.005), non‐smokers (GG vs. AA/GG: adjusted OR = 2.02, 95% CI: 1.07–3.78, *P* = 0.029), and nondrinkers (GG vs. AA/GG: adjusted OR = 1.98, 95% CI: 1.07–3.66, *P* = 0.029) (Table 4).

**Table 4:** Stratified analyses between *CASC8* rs1562430 A > G polymorphism and ESCC risk by sex, age, smoking status, and alcohol consumption

	*CASC8* rs1562430 A > G (case/control)^†^				Adjusted OR^‡^ (95% CI); *P*‐value				
Variable	AA	AG	GG	AG + GG	AA	AG	GG	AG + GG	GG vs. (AG + AA)
Sex									
Male	457/611	161/261	24/15	185/276	1.00	0.80 (0.64–1.01); *P*: 0.065	**2.47 (1.27–4.81)**; ***P*: 0.008**	0.88 (0.71–1.11); *P*: 0.285	**2.62 (1.35–5.10)**; ***P*: 0.005**
Female	188/316	89/129	7/9	96/138	1.00	1.16 (0.84–1.61); *P*: 0.368	1.31 (0.48–3.58); *P*: 0.599	1.17 (0.85–1.61); *P*: 0.329	1.25 (0.46–3.41); *P*: 0.660
Age									
<63	322/496	117/207	22/15	139/222	1.00	0.84 (0.64–1.10); *P*: 0.213	**2.50 (1.26–4.95)**; ***P*: 0.009**	0.95 (0.73–1.23); *P*: 0.677	**2.62 (1.33–5.17)**; ***P*: 0.005**
≥63	323/431	133/183	9/9	142/192	1.00	0.97 (0.74–1.27); *P*: 0.840	1.41 (0.55–3.61); *P*: 0.473	0.99 (0.76–1.29); *P*: 0.956	1.42 (0.56–3.63); *P*: 0.460
Smoking status									
Never	368/648	143/261	21/20	164/281	1.00	0.94 (0.74–1.20); *P*: 0.632	**1.98 (1.05–3.73)**; ***P*: 0.034**	1.01 (0.80–1.28); *P*: 0.914	**2.02 (1.07–3.78)**; ***P*: 0.029**
Ever	277/279	107/129	10/4	117/133	1.00	0.85 (0.62–1.15); *P*: 0.282	2.51 (0.78–8.10); *P*: 0.125	0.90 (0.66–1.21); *P*: 0.471	2.64 (0.82–8.45); *P*: 0.104
Alcohol consumption									
Never	448/738	179/293	22/21	201/314	1.00	0.99 (0.80–1.24); *P*: 0.946	**1.98 (1.07–3.66)**; ***P*: 0.031**	1.05 (0.85–1.31); *P*: 0.637	**1.98 (1.07–3.66)**; ***P*: 0.029**
Ever	197/189	71/97	9/3	80/100	1.00	0.72 (0.50–1.03); *P*: 0.074	2.73 (0.73–10.25); *P*: 0.138	0.78 (0.54–1.11); *P*: 0.170	3.01 (0.80–11.27); *P*: 0.102

^†^ The genotyping was successful in 926 (97.6%) ESCC cases, and 1341 (98.0%) controls for *CASC8* rs1562430 A > G.

^‡^ Adjusted for age, sex, smoking status and alcohol consumption (besides stratified factors accordingly) in a logistic regression model.

## Discussion

There are many possible causes of ESCC including both environmental and genetic factors. In the present study, we investigated the association between SNPs in the lncRNA *CASC8* gene and susceptibility to ESCC. We found that rs1562430 was significantly associated with increased risk of ESCC. In stratification analyses, we found that the increased ESCC risk was significantly associated with CASC8 rs1562430 GG genotype among subjects for males, never‐drinkers, never‐smokers and those age < 60.

The CASC8 gene is located at 8q24 and has no translation capabilities; however, it can affect the progression of the disease by regulating the function of the coding region.^29^ *CASC8* gene polymorphisms play important roles in different cancers.^21^, ^22^, ^23^, ^24^ Furthermore, the lncRNA *CASC8* suppresses the proliferation of bladder cancer cells by downregulating glycolysis.^30^ The results of this study suggested that the *CASC8* SNP rs1562430 could be a predictive biomarker for susceptibility to ESCC.

The *CASC8* rs10505477 variant is related to the development of numerous cancers including colorectal,^31^ gastric,^32^ and ovarian cancers.^33^ Zhang *et al*. reported that the SNP rs6983267 GG genotype was significantly associated with increased lung cancer risk in Han Chinese.^34^ In the current study, we used the *CASC8* rs1562430 AA genotype as the reference group and found that the GG genotype can significantly increase the risk of ESCC.

The present study had several limitations, and further investigation is necessary to resolve these deficiencies. First, we had large geographical restrictions on the size and scope of the sample, and the sample size was insufficient. Second, in the hierarchical analysis, limitations in basic information of patients and control populations prevented analysis of gene‐environment or gene‐gene interactions, which may influence ESCC risk. Third, the heterogeneity of the population was limited; therefore, other SNPs with significant effects may have remained undetected.

In summary, we explored the effect of the *CASC8* rs1562430 polymorphism on ESCC susceptibility and found that functional polymorphisms in *CASC8* rs1562430 A > G may affect individual susceptibility to ESCC. A larger sample size and multidimensional information are needed to validate our results.

## Disclosure

All authors declared no conflict of interest.
